# Evaluation of a 55-gene classifier as a prognostic biomarker for adjuvant chemotherapy in stage III colon cancer patients

**DOI:** 10.1186/s12885-021-09088-6

**Published:** 2021-12-14

**Authors:** Eiji Oki, Eiji Shinto, Mototsugu Shimokawa, Shigeki Yamaguchi, Megumi Ishiguro, Seiji Hasegawa, Yasumasa Takii, Hideyuki Ishida, Tetsuya Kusumoto, Masaru Morita, Naohiro Tomita, Manabu Shiozawa, Masafumi Tanaka, Heita Ozawa, Yojiro Hashiguchi, Shinobu Ohnuma, Sachiyo Tada, Tomoko Matsushima, Kazuo Hase

**Affiliations:** 1 Department of Surgery and Science, Graduate School of Medical Sciences, Kyushu University, 3-1-1, Maidashi, Higashi-ku, Fukuoka, 812-8582 Japan; 2 Department of Surgery, National Defense Medical College, Tokorozawa, Japan; 3 Clinical Research Institute, National Hospital Organization Kyushu Cancer Center, Fukuoka, Japan; 4 Department of Gastroenterological Surgery, Saitama Medical University International Medical Center, Hidaka, Japan; 5 Department of Translational Oncology, Graduate School of Medical and Dental Science, Tokyo Medical and Dental University, Tokyo, Japan; 6 Department of Surgery, Saiseikai Yokohamashi Nanbu Hospital, Yokohama, Japan; 7 Department of Gastroenterological Surgery, Niigata Cancer Center Hospital, Niigata, Japan; 8 Department of Digestive Tract and General Surgery, Saitama Medical Center, Saitama Medical University, Kawagoe, Japan; 9 Department of Gastroenterological Surgery, Clinical Research Center, Cancer Research Division, National Hospital Organization Kyushu Medical Center, Fukuoka, Japan; 10 Gastroenterological Surgery, National Hospital Organization Kyushu Cancer Center, Fukuoka, Japan; 11 Department of Surgery, Hyogo College of Medicine, Nishinomiya, Japan; 12 Colorectal Surgery Division, Kanagawa Cancer Center, Yokohama, Japan; 13 Coloproctology Center, Takano Hospital, Kumamoto, Japan; 14 Department of Colorectal Surgery, Tochigi Cancer Center, Utsunomiya, Japan; 15 Department of Surgery, Teikyo University School of Medicine, Tokyo, Japan; 16 Department of Surgery, Tohoku University Hospital, Sendai, Japan; 17 LS Business, Sysmex Corporation, Kobe, Japan

**Keywords:** Colon cancer, Predictive, Adjuvant chemotherapy, Oxaliplatin, Subtyping

## Abstract

**Background:**

Adjuvant chemotherapy reduces the risk of recurrence of stage III colon cancer (CC). However, more effective prognostic and predictive biomarkers are needed for better treatment stratification of affected patients. Here, we constructed a 55-gene classifier (55GC) and investigated its utility for classifying patients with stage III CC.

**Methods:**

We retrospectively identified patients aged 20–79 years, with stage III CC, who received adjuvant chemotherapy with or without oxaliplatin, between the years 2009 and 2012.

**Results:**

Among 938 eligible patients, 203 and 201 patients who received adjuvant chemotherapy with and without oxaliplatin, respectively, were selected by propensity score matching. Of these, 95 patients from each group were analyzed, and their 5-year relapse-free survival (RFS) rates with and without oxaliplatin were 73.7 and 77.1%, respectively. The hazard ratios for 5-year RFS following adjuvant chemotherapy (fluoropyrimidine), with and without oxaliplatin, were 1.241 (95% CI, 0.465–3.308; *P* = 0.67) and 0.791 (95% CI, 0.329–1.901; *P* = 0.60), respectively. Stratification using the 55GC revealed that 52 (27.3%), 78 (41.1%), and 60 (31.6%) patients had microsatellite instability (MSI)-like, chromosomal instability (CIN)-like, and stromal subtypes, respectively. The 5-year RFS rates were 84.3 and 72.0% in patients treated with and without oxaliplatin, respectively, for the MSI-like subtype (HR, 0.495; 95% CI, 0.145–1.692; *P* = 0.25). No differences in RFS rates were noted in the CIN-like or stromal subtypes. Stratification by cancer sidedness for each subtype showed improved RFS only in patients with left-sided primary cancer treated with oxaliplatin for the MSI-like subtype (*P* = 0.007). The 5-year RFS rates of the MSI-like subtype in left-sided cancer patients were 100 and 53.9% with and without oxaliplatin, respectively.

**Conclusions:**

Subclassification using 55GC and tumor sidedness revealed increased RFS in patients within the MSI-like subtype with stage III left-sided CC treated with fluoropyrimidine and oxaliplatin compared to those treated without oxaliplatin. However, the predictive power of 55GC subtyping alone did not reach statistical significance in this cohort, warranting larger prospective studies.

**Trial registration:**

The study protocol was registered in the University Hospital Medical Education Network (UMIN) clinical trial registry (UMIN study ID: 000023879).

**Supplementary Information:**

The online version contains supplementary material available at 10.1186/s12885-021-09088-6.

## Background

Colorectal cancer remains one of the most common causes of cancer-related mortality worldwide [1]. Adjuvant chemotherapy in stage III colon cancer (CC) after curative intent resection prolongs survival and reduces the risk of tumor recurrence [2]. Pivotal trials have shown superior outcomes for fluoropyrimidine in combination with oxaliplatin compared with fluoropyrimidine alone in most patient populations; however, the evidence is less well established in elderly patients. Emerging data have also led to a debate over the optimal duration of chemotherapy, specifically in the context of increased toxicity [3]. Furthermore, subclassification of stage III CC is an ongoing process based on accumulating patient survival data and features of cancer presentation [4]. Therefore, better prognostic and predictive biomarkers are required to stratify patients for adjuvant therapies based on chemotherapy regimen and duration.

The consensus molecular subtype (CMS) is a robust classification system of colorectal cancer types based on over 600 genes. DNA microarray analysis of CMS can provide a valuable prognostic information [5]. CMS is also potentially predictive, as different subtypes vary in sensitivity to adjuvant chemotherapy. We previously simplified this classification by constructing a 55-gene classifier (55GC), focusing on genes located on the long arms of chromosomes 18 and 20, as well as on stroma-related genes [6]. Using the 55GC, we categorized stage II/III CC into three subtypes with different recurrence rates: “microsatellite instability (MSI)-like,” “chromosomal instability (CIN)-like,” and “stromal” subtypes and showed a prognostic utility of such a system in a single institutional study. We conducted a validation study using a 55-gene classifier to assess stratification recurrence (55 STAR) risk. Furthermore, 55GC-based subtyping was able to stratify stage II CC recurrence risk in a multi-institutional validation cohort study of 232 patients [7]. To expand on these previous findings, we hypothesized that 55GC could be utilized to stratify survival of patients with stage III CC who receive adjuvant chemotherapy with or without oxaliplatin.

## Methods

### Tissue samples

**Fig. 1:**
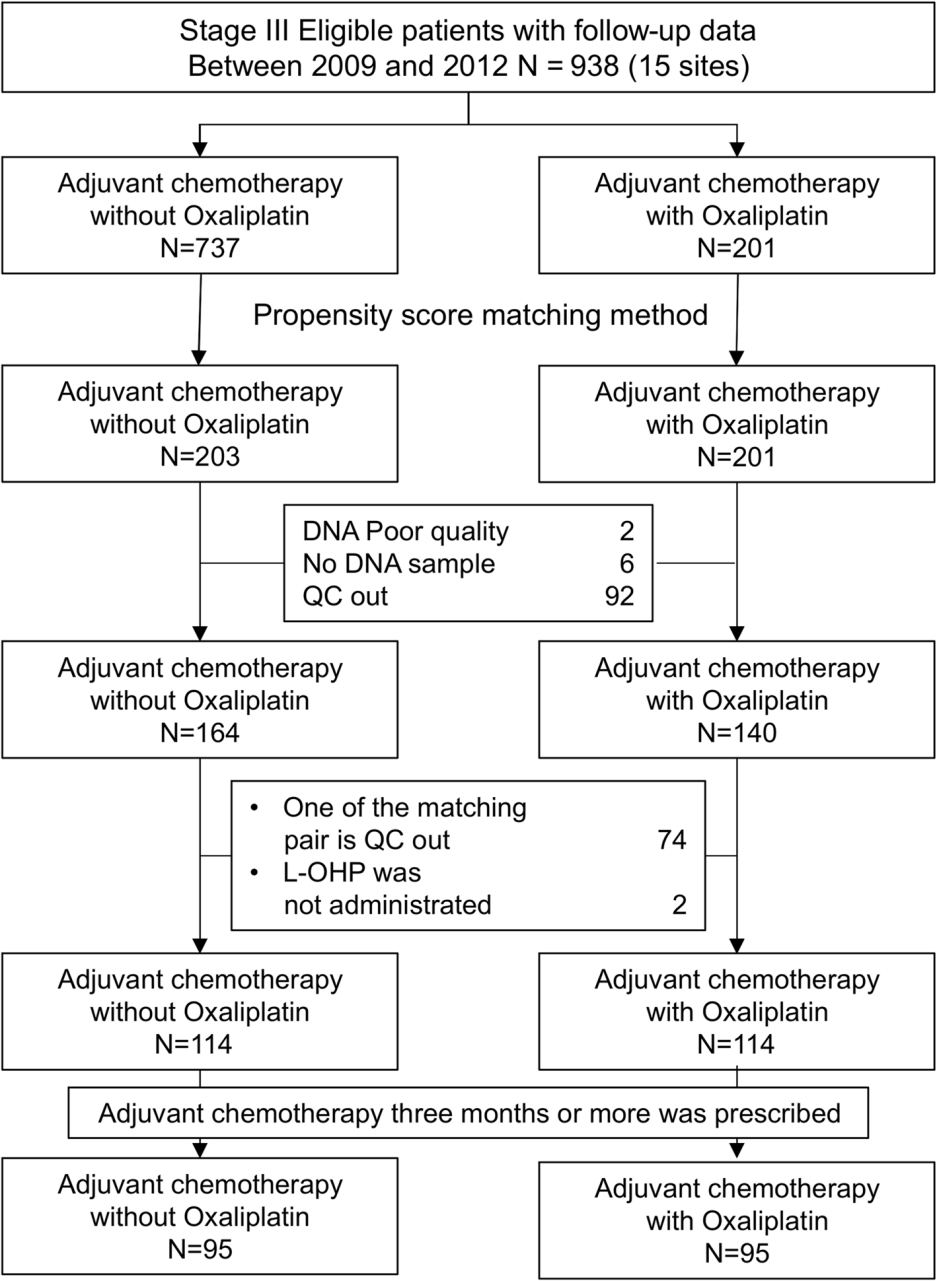
CONSORT diagram

We retrospectively identified consecutive patients with stage III colon and rectosigmoid CC aged 20–79 years who underwent curative surgery (R0) and received adjuvant chemotherapy with or without oxaliplatin from 15 institutions in Japan between January 1, 2009, and December 31, 2012. Patients who received neoadjuvant treatment, had multiple active cancers, died, or had recurrence within 60 days post-surgery, were excluded from the study. Relevant patient characteristics were recorded. The study protocol was approved by the institutional review boards of Kyushu University (study ID 28–69), National Defense Medical College (study ID 2477), Saitama Medical University International Medical Center (study ID 16–051), Tokyo Medical and Dental University (study ID G2016–007), Saiseikai Yokohamashi Nanbu Hospital (study ID 2017-D21), Niigata Cancer Center Hospital (study ID 796), Saitama Medical University Saitama Medical Center (study ID 1812), National Hospital Organization Kyushu Medical Center (study ID 16C058), National Hospital Organization Kyushu Cancer Center (study ID 2016–48), Hyogo College of Medicine (study ID Hi326), Kanagawa Cancer Center (study ID 2017–8), Takano Hospital (study ID 16–04), Tochigi Cancer Center (study ID A432), Teikyo University School of Medicine (study ID 16–057), Tohoku University (study ID 2016–1-222) and Sysmex Corporation (study ID 2015–71), and was registered in the University Hospital Medical Education Network Clinical Trial Registry (UMIN study ID 000023879). All procedures were carried out in accordance with the relevant guidelines and regulations. Because this study was a retrospective observational study carried out in Japan, informed consent was obtained using the opt-out/opt-in approach, according to each participating institution’s policy (as per Japanese ethical guidelines for an observational study, consent of the family is not required for dead participants). The Consolidated Standards of Reporting Trials (CONSORT) diagram is shown in Fig. 1.

### Gene expression analysis

Formalin-fixed paraffin-embedded (FFPE) primary cancer tissue specimens containing the invasive tumor front with the greatest depth of invasion were collected from each institution, and a single 5-μm section was sent to the Takeda Pathology Center (Osaka, Japan) for analysis. Total RNA was extracted from fewer than four unstained 10-μm sections for gene expression microarray assay profiling using the RNeasy FFPE Kit (Qiagen, Valencia, CA, USA). Samples with insufficient RNA quality for microarray analysis were excluded from this cohort. Gene expression data were generated using the Affymetrix GeneChip Human Genome U133 Plus 2.0 Array (Thermo Fisher Scientific; Waltham, MA) and analyzed using the 55GC model as described previously [6]. DNA was extracted using the QIAamp DNA FFPE Tissue Kit (Qiagen). *RAS* mutations were assessed using the MEBGEN RASKET KIT (Medical & Biological Laboratories; Nagoya, Japan).

### Statistical analyses

The primary endpoint was RFS, defined as the time from surgery to the first CC recurrence or death from any cause. A propensity score method was used to reduce the selection bias, and a logistic regression model was used to calculate patient propensity scores. Propensity score matching was performed for the number of lymph node metastases, tumor location, sex, and age in a 1:1 ratio using a caliper width of 0.1. Demographic characteristics are summarized using contingency tables. The RFS curve was estimated using the Kaplan–Meier method and compared between groups using log-rank tests. HRs and 95% CIs were calculated using the Cox proportional hazards model. Risk factors for RFS were assessed using a Cox proportional hazards model with a backward elimination method that included known clinicopathological prognostic factors and gene mutations as covariates. Subgroup analysis was performed for age (< 70 vs. ≥70 years), sex (male vs. female), carcinoembryonic antigen (<upper limit of normal [ULN] vs. ≥ULN), tumor location (left vs. right), T stage (T1–T3 vs. T4), lymph node metastasis (N1 vs. N2–N3), tumor grade (poorly differentiated and mucinous adenocarcinoma vs. tubular adenocarcinoma), vascular invasion (v0 vs. v1–v3), subtype (CIN vs. MSI vs. stromal), and *RAS* status (wild vs. mutant). Fisher’s exact test was used to compare patient characteristics between the groups. *P*-values were two-sided, and statistical significance was set at *P* < 0.05. All statistical analyses were performed using the Statistical Analysis System, version 9.4 (SAS Institute, Cary, NC).

## Results

### Patient characteristics

**Fig. 2:**
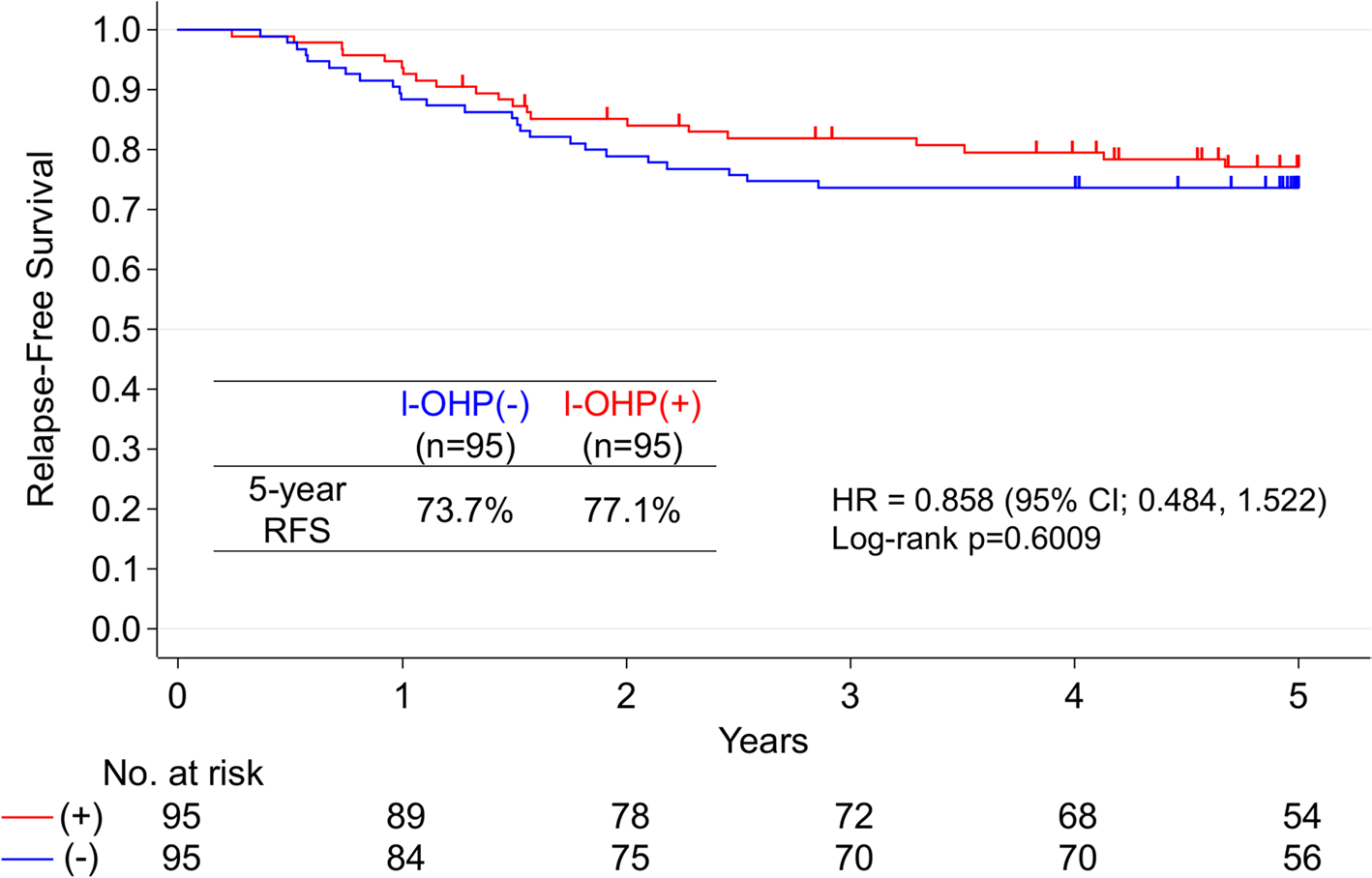
Five-year relapse-free survival (RFS) curves of all patients treated with [I-OHP(+), indicated in red] and without oxaliplatin [I-OHP(−), indicated in blue]

Among 938 eligible patients, 203 and 201 individuals receiving adjuvant chemotherapy with and without oxaliplatin, respectively, were selected using propensity score matching. After excluding patients with low-quality specimens and those who had received chemotherapy for < 3 months, 95 patients from each group were analyzed (Fig. 1). In the overall cohort of 190 patients (Table 1), 98 (51.6%) patients were men, 146 (76.8%) were aged < 70 years, and 126 (66.3%) had left-sided tumors. Regarding histopathological characteristics, there were more patients with T4 stage cancer in the cohort treated with oxaliplatin (44/95 patients, 46.3%) than in the cohort treated without oxaliplatin (34/95 patients, 35.8%) (*P* = 0.022). In addition, there were more patients with < 12 resected lymph nodes in the cohort treated with oxaliplatin (15/95 patients, 15.8%) than in the cohort treated without oxaliplatin (5/95 patients, 5.3%) (*P* = 0.018). The 5-year RFS rates were 73.7 and 77.1% in patients treated with and without oxaliplatin, respectively (Fig. 2; hazard ratio [HR]: 0.858; 95% confidence interval [CI]: 0.484–1.522).

**Table 1:** Patient characteristics

Factors		Oxaliplatin (−) (*N* = 95)	Oxaliplatin (+) (*N* = 95)	Total (*N* = 190)	*P* value
		n (%)	n (%)	n (%)	
Sex	Male	46 (48.4)	52 (54.7)	98 (51.6)	0.3838
	Female	49 (51.6)	43 (45.3)	92 (48.4)	
Age (years)	< 70	71 (74.7)	75 (78.9)	146 (76.8)	0.4915
	≥70	24 (25.3)	20 (21.1)	44 (23.2)	
CEA	<ULN	63 (66.3)	50 (52.6)	113 (59.5)	0.6105
	≥ULN	31 (32.6)	20 (21.1)	51 (26.8)	
	Unknown	1 (1.1)	25 (26.3)	26 (13.7)	
Tumor location	Right side	31 (32.6)	33 (34.7)	64 (33.7)	0.7588
	Left side	64 (67.4)	62 (65.3)	126 (66.3)	
T stage	T1-T3	61 (64.2)	51 (53.7)	112 (58.9)	0.1403
	T4	34 (35.8)	44 (46.3)	78 (41.1)	
Tumor grade	por & muc	4 (4.2)	13 (13.7)	17 (8.9)	0.0222
	tub	91 (95.8)	82 (86.3)	173 (91.1)	
Lymphatic invasion	Negative	29 (30.5)	26 (27.4)	55 (28.9)	0.6313
	Positive	66 (69.5)	69 (72.6)	135 (71.1)	
Vascular invasion	Negative	28 (29.5)	23 (24.2)	51 (26.8)	0.4130
	Positive	67 (70.5)	72 (75.8)	139 (73.2)	
N stage	N1	39 (41.1)	36 (37.9)	75 (39.5)	0.0940
	N2	54 (56.8)	50 (52.6)	104 (54.7)	
	N3	2 (2.1)	9 (9.5)	11 (5.8)	
Number of resected lymph nodes	< 12	5 (5.3)	15 (15.8)	20 (10.5)	0.0181
	≥12	90 (94.7)	80 (84.2)	170 (89.5)	

*CEA* Carcinoembryonic antigen; *ULN* Upper limit of normal

### 55-gene classifier subtype analysis

In the total cohort, 55GC analysis revealed 52 (27.3%) patients with an MSI-like subtype, 78 (41.1%) patients with a CIN-like subtype, and 60 (31.6%) patients with a stromal subtype. The clinicopathological characteristics of each subtype are shown in Table 2. MSI-like subtype tumors were more likely to be right-sided, whereas CIN-like subtype tumors were more likely to be left-sided compared to the overall cohort. The MSI-like subtype tumors had a higher proportion of mucinous subtype (6/52 tumors, 11.5%) compared with CIN-like (0/78 tumors, 0%) and stromal-like (2/60 tumors, 3.3%) tumors. We found no difference in lymphatic and vascular invasion between the three subtypes. However, higher proportions of N2/N3 (39/52 tumors, 75.0%) in MSI-like tumors were found compared to the other subtypes.

**Table 2:** Patient characteristics by tumor subtype

Factors		MSI-like (***N*** = 52) n (%)	CIN-like (***N*** = 78) n (%)	Stromal (***N*** = 60) n (%)	Total (***N*** = 190) n (%)	***P*** value
Sex	Male	28 (53.8)	39 (50.0)	31 (51.7)	98 (51.6)	*p* = 0.9116
	Female	24 (46.2)	39 (50.0)	29 (48.3)	92 (48.4)	
Age	< 70	37 (71.2)	65 (83.3)	44 (73.3)	146 (76.8)	*p* = 0.2011
	≥70	15 (28.8)	13 (16.7)	16 (26.7)	44 (23.2)	
CEA	<ULN	33 (63.5)	51 (65.4)	29 (48.3)	113 (59.5)	*p* = 0.1847
	≥ULN	14 (26.9)	17 (21.8)	20 (33.3)	51 (26.8)	
	Unknown	5 (9.6)	10 (12.8)	11 (18.3)	26 (13.7)	
Tumor location	Right side	26 (50.0)	18 (23.1)	20 (33.3)	64 (33.7)	*p* = 0.0063
	Left side	26 (50.0)	60 (76.9)	40 (66.7)	126 (66.3)	
T stage	T1-T3	30 (57.7)	50 (64.1)	32 (53.3)	112 (58.9)	*p* = 0.4335
	T4	22 (42.3)	28 (35.9)	28 (46.7)	78 (41.1)	
Histology	Non muc	46 (88.5)	78 (100.0)	58 (96.7)	182 (95.8)	*p* = 0.0036
	muc	6 (11.5)	0 (0.0)	2 (3.3)	8 (4.2)	
Tumor grade	por & muc	11 (21.2)	2 (2.6)	4 (6.7)	17 (8.9)	*p* = 0.0010
	tub	41 (78.8)	76 (97.4)	56 (93.3)	173 (91.1)	
Lymphatic invasion	Negative	14 (26.9)	28 (35.9)	13 (21.7)	55 (28.9)	*p* = 0.1754
	Positive	38 (73.1)	50 (64.1)	47 (78.3)	135 (71.1)	
Vascular invasion	Negative	14 (26.9)	26 (33.3)	11 (18.3)	51 (26.8)	*p* = 0.1433
	Positive	38 (73.1)	52 (66.7)	49 (89.7)	139 (73.2)	
N stage	N1	13 (25.0)	39 (50.0)	23 (38.3)	75 (39.5)	*p* = 0.0021
	N2	34 (65.4)	39 (50.0)	31 (51.7)	104 (54.7)	
	N3	5 (9.6)	0 (0.0)	6 (10.0)	11 (5.8)	
Number of resected lymph nodes	< 12	3 (5.8)	11 (14.1)	6 (10.0)	20 (10.5)	*p* = 0.3125
	≥12	49 (94.2)	67 (85.9)	54 (90.0)	170 (89.5)	
Adjuvant chemotherapy	With oxaliplatin	25 (48.1)	42 (53.8)	28 (46.7)	95 (50.0)	*p* = 0.6686
	Without oxaliplatin	27 (51.9)	36 (46.2)	32 (53.3)	95 (50.0)	

*CEA* Carcinoembryonic antigen; *ULN* Upper limit of normal; *muc* Mucinous; *por & muc* Poorly differentiated and mucinous adenocarcinoma; *tub* Tubular adenocarcinoma

### Survival analysis according to the 55-gene classifier analysis and chemotherapy regimen

**Fig. 3:**
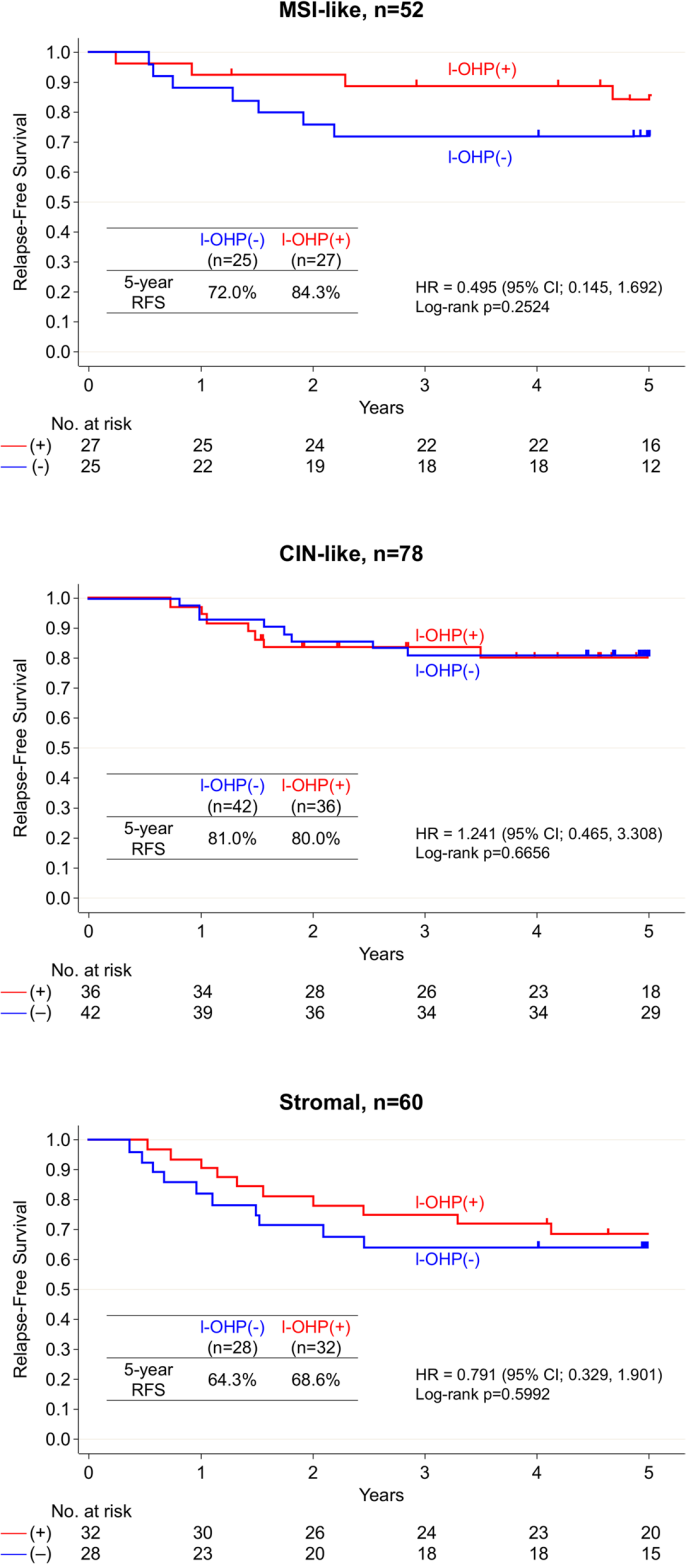
Five-year relapse-free survival (RFS) curves of patients treated with [I-OHP(+), indicated in red] and without oxaliplatin [I-OHP(−), indicated in blue] according to the 55-gene classifier (55GC) subtypes [top: microsatellite instability (MSI)-like; middle: chromosomal instability (CIN)-like; bottom: stromal]

**Fig. 4:**
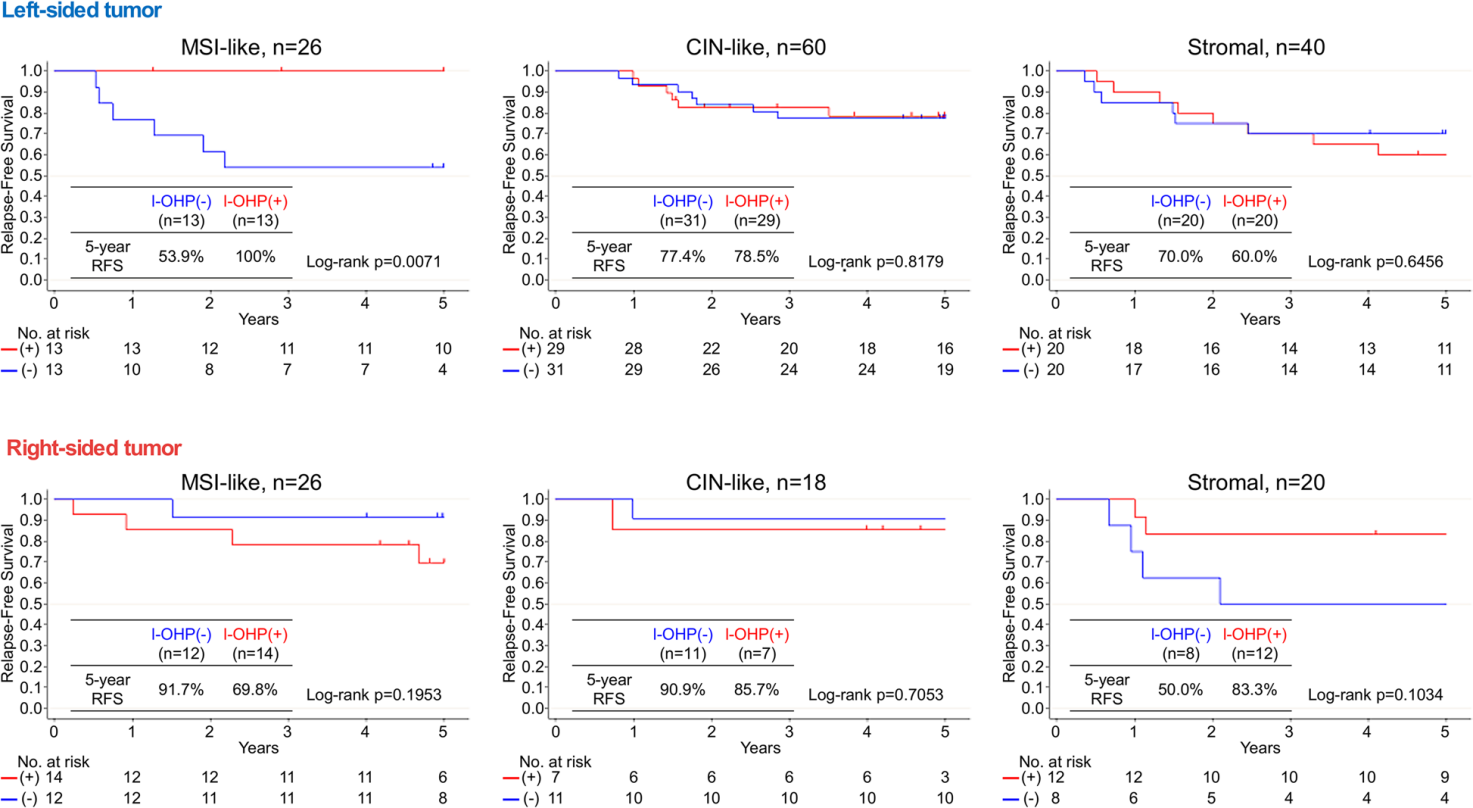
Five-year relapse-free survival (RFS) curves of patients treated with [I-OHP(+), indicated in red] and without oxaliplatin [I-OHP(−), indicated in blue] according to the 55-gene classifier (55GC) subtypes and primary tumor sidedness (top: left-side; bottom: right-side)

Comparisons of RFS in patients treated with and without oxaliplatin according to the 55GC subtype are shown in Fig. 3. The 5-year RFS rates were 84.3 and 72.0% in patients treated with and without oxaliplatin, respectively, for the MSI-like subtype (HR, 0.495; 95% CI, 0.145–1.692); however, the trend was not statistically significant (log-rank *P* = 0.25). There was no difference in RFS in CIN-like subtype patients according to oxaliplatin treatment status (HR, 1.241; 95% CI, 0.465–3.308; log-rank *P* = 0.67). RFS was also unchanged in the stromal subtype patients regardless of oxaliplatin treatment (HR, 0.791; 95% CI, 0.329–1.901; log-rank *P* = 0.60). Further subdivision into left- and right-sided primary cancer of the subtypes showed improved RFS only for left-sided primary cancer of the MSI-like subtype treated with oxaliplatin (Fig. 4; log-rank *P* = 0.0071). The 5-year RFS rates for the MSI-like subtype in left-sided cancer were 100 and 53.9% with and without oxaliplatin, respectively. No significant differences in RFS were noted between subtypes when stratified by treatment with (log-rank *P* = 0.23) and without oxaliplatin (log-rank *P* = 0.37; Additional file 1).

Subgroup analysis is shown in Additional file 2. Comparison of RFS in patients treated with or without oxaliplatin revealed no significant differences according to patient characteristics (age, sex) or histopathological findings (tumor location, T stage, N stage, tumor grade, and vascular invasion).

## Discussion

Despite a significant progress in the development of prognostic and predictive biomarkers for CC, particularly the *RAS* mutation status and deficient mismatch repair (dMMR) status to guide therapy for a metastatic disease [8, 9], there is an ongoing need for better tools enabling molecular analysis of early stage CC to guide adjuvant therapy. While dMMR status may indicate a lack of efficacy of fluoropyrimidine-only regimens without oxaliplatin, there is a lack of validated predictive tumor biomarkers for early stage CC [10, 11]. Several multigene expression profiling systems, such as Oncotype DX (Genomic Health, Redwood City, CA) and ColoPrint (Agendia; Amsterdam, Netherlands), have been developed [12]. However, they are not subtyping systems; therefore, they have a prognostic but no predictive value for chemosensitivity. In contrast, the predictive potential of molecular subtypes in CC has recently been demonstrated in prospective trials [13–15]. While the classification of CMS is considered the most robust classification based on comprehensive gene expression profiling [5], other classifications have been developed and validated [16–19]. However, optimal methods of subtype identifications and difficulties in the practical widespread measurement of these genotypes in routine clinical practice are the subject of a heated debate [20]. Recently, other gene set classifiers obtained from a microarray analysis used in CMS have been reported. A similar prognostic utility was shown using 99 or 200 gene sets [21]. In this study, we present a potential utility of a 55 gene set, especially when it is accompanied by the assessment of other cancer properties, such as sidedness. The method may provide a prognostic and predictive information for guiding adjuvant therapy in early stage CC after curative-intent resection.

Post-hoc analyses of tumor tissue from patients in large randomized trials of adjuvant chemotherapy have revealed the overall poor prognosis of certain molecular subtypes. For example, analysis of the National Surgical Adjuvant Breast and Bowel Project (NSABP) C-07 clinical trial demonstrated poor prognosis in both stage II and III patients with a ‘stem-like’ subtype identified from three different subtyping methods [22]. This is consistent with our findings, which show a tendency for poorer prognosis of the stromal subtype in stage III patients, as well as our previous results regarding stage II/III CC patients [6]. Furthermore, in the aforementioned NSABP C-07 retrospective analysis, the stem-like subtype from the Colorectal Cancer Assigner classification (CRCA) predicted a lack of benefit for the addition of oxaliplatin [22]. Similar to the NSABP C-07 study, our cohort demonstrated a 3.4% improvement in 5-year RFS due to the addition of oxaliplatin to adjuvant chemotherapy. Considering this observation, the potential additive benefit of oxaliplatin for adjuvant chemotherapy in the MSI-like subtype from our 55GC system requires further investigation and validation. Similar to the CMS classification, the 55GC system requires caution; the MSI-like subtype is not identical to the MSI-high or dMMR CC. In our cohort, MSI-like subtype tumors had a higher proportion of mucinous tumors and higher proportions of N2/N3 cases compared to the other subtypes. Previous reports have shown that MSI-H tumors have a higher proportion of mucinous subtypes and a lower proportion of lymph node metastasis [23, 24]. One of the reasons why the MSI-H subtype tends to be associated with good prognosis is that this subtype rarely involves lymph node metastasis. Our data showed that the MSI-H like subtype is quite different from the MSI-H and MMR subtypes.

Similar to the CMS classification, our classification showed the potential additive benefit of oxaliplatin for adjuvant chemotherapy in the MSI-like subtype. Recently, circulating tumor DNA (ctDNA) has been shown to be a promising and accurate predictive marker for tumor recurrence [25, 26]. Treatment of ctDNA-positive patients with aggressive chemotherapy may therefore reduce recurrence rates. In line, we initiated a nationwide large-scale clinical trial named CIRCULATE-Japan [27], which consists of a prospective observational study and two accompanying interventional studies to elucidate the predictive value of ctDNA for the recurrence risk. ctDNA may be a strong predictive marker for recurrence; however, it could not reveal the intrinsic subtype of cancer. Therefore, an optimized combination of a few prognostic methods will probably be utilized in the clinic in the future.

The major limitation of the current study is the relatively small number of patients in each analyzed subgroup, especially after accounting for propensity score matching. Nevertheless, the results suggest the potential benefit of adjuvant chemotherapy with oxaliplatin in the MSI-like cancer subtype when accounting for tumor sidedness. These findings require further prospective validation in an independent cohort to determine their true clinical significance. Combined with deeper analysis of genomic and histopathologic correlates, including the immune infiltrate and tumor microenvironment, our data could improve our understanding of the biological underpinnings of each tumor subtype, resulting in more accurate diagnosis and treatment stratification.

## Conclusions

In conclusion, the current 55GC study highlights that oxaliplatin may have an additive effect in adjuvant chemotherapy for the MSI-like CC subtype, especially for left-sided primary tumors. Hence, future studies with larger numbers of CC cases are warranted to validate our findings.

## Supplementary Information

**Additional file 1: Figure S1.** Five-year relapse-free survival (RFS) curves in the 55-gene classifier (55GC) subtypes according to the adjuvant chemotherapy received [blue: microsatellite instability (MSI)-like; red: chromosomal instability (CIN)-like; green: stromal].

**Additional file 2: Figure S2.** Subgroup analysis.

## Data Availability

The cDNA microarray datasets generated during the current study are available from the corresponding author upon a reasonable request. The data were not reposited in the Gene Expression Omnibus (GEO) database due to concerns related to the Japanese Act of the Protection of Personal Information.

## Abbreviations

55GC: 55 gene classifier
CC: Colon cancer
CEA: Carcinoembryonic antigen
CI: Confidence interval
CIN: Chromosomal instability
CMS: Consensus molecular subtypes
CONSORT: Consolidated Standards of Reporting Trials
CRCA: Colorectal Cancer Assigner
DNA: Deoxyribonucleic acid
FFPE: Formalin-fixed paraffin-embedded
HR: Hazard ratio
MSI: Microsatellite instability
NSABP: National Surgical Adjuvant Breast and Bowel Project
*P*: *P*-value
RFS: Relapse-free survival
RNA: Ribonucleic acid
STAR: Stratification recurrence
ULN: Upper limit of normal
UMIN: University Hospital Medical Education Network
