# Supplementary material for: Evaluation of a 55-gene classifier as a prognostic biomarker for adjuvant chemotherapy in stage III colon cancer patients
Source: BMC Cancer. 2021 Dec 14;21:1332. doi: 10.1186/s12885-021-09088-6 (PMC8672629; doi:10.1186/s12885-021-09088-6)
Supplement: Supplementary file 1 — Additional file 1: Figure S1. Five-year relapse-free survival (RFS) curves in the 55-gene classifier (55GC) subtypes according to the adjuvant chemotherapy received [blue: microsatellite instability (MSI)-like; red: chromosomal instability (CIN)-like; green: stromal]. [file 12885_2021_9088_MOESM1_ESM.docx]

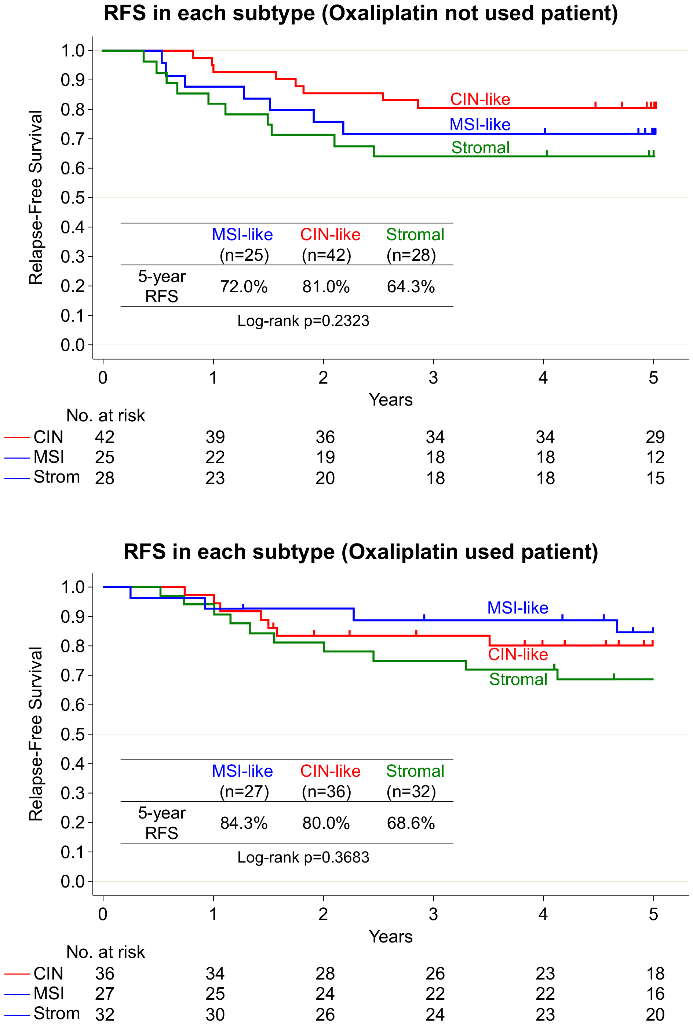


**Figure S1**. Five-year relapse-free survival (RFS) curves in the 55-gene classifier (55GC) subtypes according to the adjuvant chemotherapy received [blue: microsatellite instability (MSI)-like; red: chromosomal instability (CIN)-like; green: stromal].
