# Supplementary figures and images for: Evaluation of a 55-gene classifier as a prognostic biomarker for adjuvant chemotherapy in stage III colon cancer patients
Source: BMC Cancer. 2021 Dec 14;21:1332. doi: 10.1186/s12885-021-09088-6 (PMC8672629; doi:10.1186/s12885-021-09088-6)

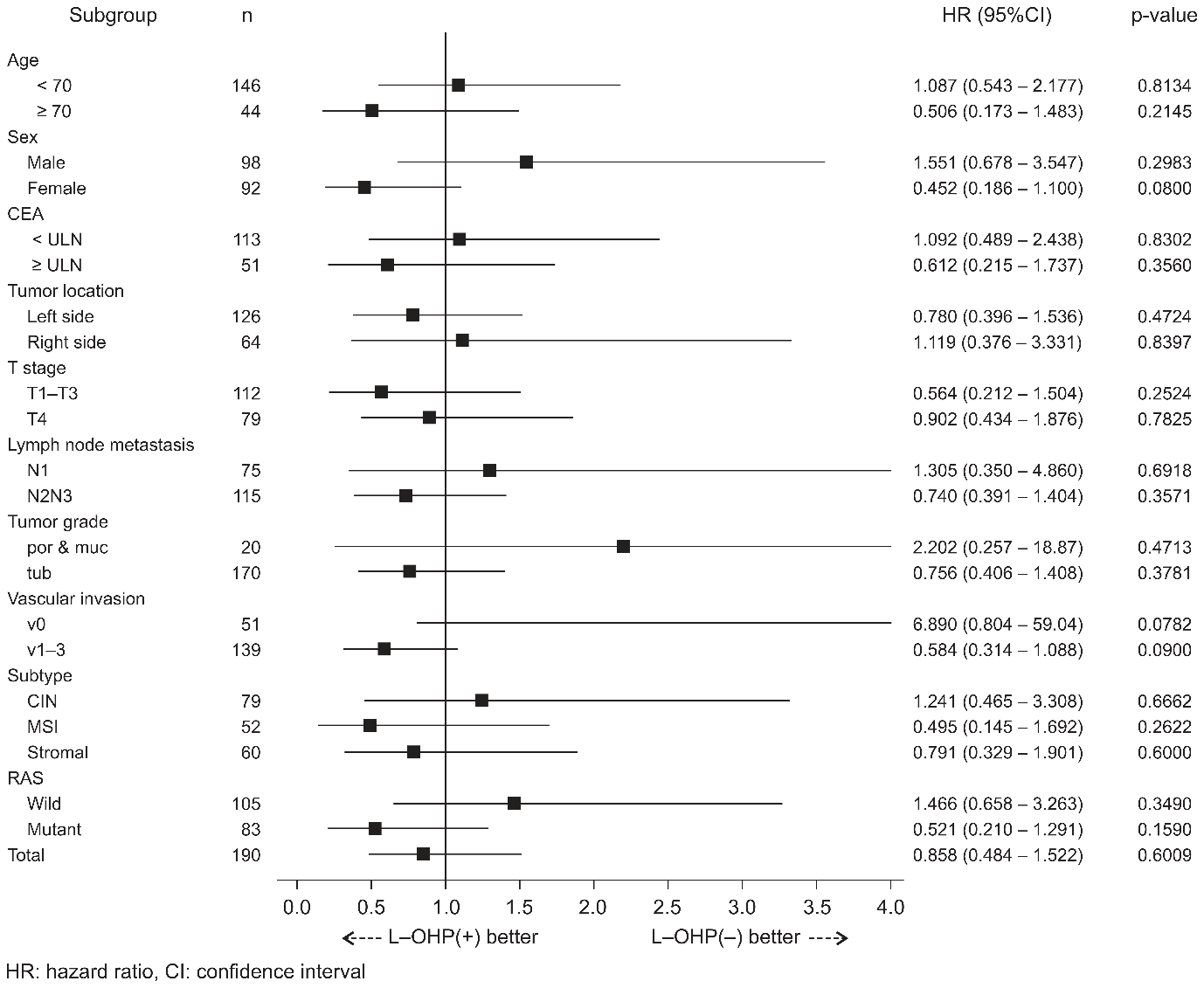


**Figure S2**. Subgroup analysis

Supplement: Supplementary file 2 — Additional file 2: Figure S2. Subgroup analysis. [file 12885_2021_9088_MOESM2_ESM.docx]
